# Feasibility and effectiveness of distal radial access in ST-elevation myocardial infarction from a SPEEDY PCI subanalysis

**DOI:** 10.1038/s41598-026-40017-4

**Published:** 2026-02-17

**Authors:** Akihiko Takahashi, Sho Torii, Yujiro Ono, Masanori Taniwaki, Mitsutoshi Oguri, Masanori Teramura, Ryuichi Kato, Shuji Otsuki, Hiroshi Suzuki, Fuminobu Yoshimachi, Hironori Ueda, Keisuke Shioji, Gaku Nakazawa, Kaoru Sakurai, Mitsuru Tsujimoto, Motosu Ando, Toshiyuki Kozai, Rie Aoyama, Yuji Ikari

**Affiliations:** 1 Department of Cardiology, Sakurakai Takahashi Hospital, 5-18-1 Oikecho, Sumaku, Kobe, 654-0026 Japan; 2 Department of Cardiology, Tokai University School of Medicine, Isehara, Japan; 3 Department of Cardiology, NHO Higashihiroshima Medical Center, Higashihiroshima, Japan; 4 Department of Cardiology, Tokorozawa Heart Center, Tokorozawa, Japan; 5 Department of Cardiology, Kasugai Municipal Hospital, Kasugai, Japan; 6 Department of Cardiology, Ichinomiya Nishi Hospital, Ichinomiya, Japan; 7 Department of Cardiology, Social Medical Corporation Yamatokai Foundation Higashiyamato Hospital, Higashiyamato, Japan; 8 Department of Cardiology, Sonoda Daiichi Hospital, Tokyo, Japan; 9 Department of Cardiology, Showa Medical University, Fujigaoka hospital, Yokohama, Kanagawa Japan; 10 Department of Cardiology, Tokai University Hachioji Hospital, Hachioji, Japan; 11 Department of Cardiology, Hiroshima Prefectural Hospital, Hiroshima, Japan; 12 Department of Cardiology, Kishiwada City Hospital, Kishiwada, Japan; 13 Department of Cardiology, Kindai University Hospital, Sakai, Japan; 14 Department of Cardiology, Shinyurigaoka General Hospital, Kawasaki, Japan; 15 Department of Cardiology, The Veritas Hospital, Kawanishi, Japan; 16 Department of Cardiology, Okamura Memorial Hospital, Shizuoka, Japan; 17 Department of Cardiology, Munakata Suikokai General Hospital, Fukutsu, Japan; 18 Department of Cardiology, Funabashi Municipal Medical Center, Funabashi, Japan; 19 Graduate School of Nursing, Kobe Women’s University, Kobe, Japan; 20 Graduate School of Nursing, Kobe Women’s University, Port Island Campus 4-7-2 Minatojima Minamimachi, Chuo-ku, Kobe, 650-0047 Japan

**Keywords:** Primary percutaneous coronary intervention, Distal radial approach, Ikari curve, ST-Elevation myocardial infarction, Single catheter PCI, Cardiology, Diseases, Medical research

## Abstract

The distal radial approach (dTRA) is increasingly recognized as a viable alternative to the conventional radial approach in coronary interventions. However, its utility in ST-elevation myocardial infarction (STEMI)—where rapid revascularization is critical—remains underexplored. To evaluate the feasibility and procedural characteristics of dTRA in STEMI patients undergoing primary percutaneous coronary intervention (PCI), using a prespecified subanalysis of the SPEEDY PCI study. Among 370 STEMI patients enrolled, 63 underwent PCI via dTRA and 307 via the conventional radial approach. A propensity score–matched analysis was performed using Killip class, GRACE score, and door-to-sheath time as covariates. After matching, the dTRA group had significantly shorter sheath-to-balloon (12.5 ± 11.5 vs. 19.7 ± 11.7 min, *p* = 0.002) and door-to-balloon times (50.2 ± 25.9 vs. 62.3 ± 19.9 min, *p* = 0.007). Procedural success and 30-day mortality rates were comparable between groups. dTRA appears feasible and safe for STEMI patients in high-volume centers. While shorter time metrics were observed, these may reflect institutional practices and operator experience rather than the access site alone.

## Introduction

The transradial approach (TRA) has become the preferred access route for percutaneous coronary intervention (PCI) in acute myocardial infarction, given its superiority over the transfemoral approach in reducing bleeding complications, shortening hospital stays, and improving clinical outcomes^1^. This preference is reflected in current European and American guidelines for primary PCI in patients with ST-elevation myocardial infarction (STEMI)^2,3^.

More recently, the distal radial approach (dTRA)—also known as the “snuffbox approach”—has emerged as a promising alternative to conventional radial approach. Initially appreciated for preserving the forearm radial artery, dTRA has demonstrated additional benefits, including a lower incidence of distal radial artery occlusion (RAO), fewer bleeding events, and improved patient comfort^4–7^. These advantages make dTRA particularly attractive in STEMI care, where minimizing access site complications is crucial. Alongside its growing clinical use and reported benefits, technical challenges have also been recognized, particularly concerns over puncture failure—even among experienced operators. These technical limitations may contribute to delays in revascularization, a critical concern in the time-sensitive context of STEMI. Nevertheless, it remains unclear whether the reported benefits of dTRA translate into consistent procedural or clinical improvements in routine clinical practice in STEMI.

To address this knowledge gap, we conducted a prespecified subanalysis, using data from the SPEEDY PCI study—a multicenter, prospective, randomized controlled trial that evaluated a single guiding catheter strategy for both diagnostic and therapeutic purposes in STEMI patients^8^. In this subanalysis, we evaluated the procedural feasibility and efficiency of dTRA versus conventional radial approach, with particular attention to time metrics and safety outcomes. While acknowledging the inherent limitations associated with non-randomized access site selection, these findings aim to yield clinically relevant insights into the role of dTRA in contemporary STEMI care.

## Methods

### Study design and population

This study represents a retrospective subanalysis of patient data derived from the SPEEDY PCI. Briefly, the parent SPEEDY PCI study was a physician-initiated, prospective, multicenter, open-label, randomized controlled trial conducted in Japan. The primary objective of the SPEEDY PCI study was to evaluate the potential benefit of reducing procedure time by using a single IKARI-left guiding catheter for both diagnostic angiography and therapeutic intervention^9–11^. Between November 2022 and October 2023, 430 patients were enrolled and randomly assigned across 47 centers in Japan. Ultimately, 380 eligible patients were analyzed. In the parent SPEEDY PCI study, the choice of vascular access site was left to the operator’s discretion, primarily based on procedural expertise. As a result, various access sites were used, including the forearm radial artery, distal radial artery, femoral artery, and others^8^. For the current subanalysis, patients treated via either the conventional radial approach or dTRA were selected, and clinical indices and outcomes were compared between the two groups. Because vascular access site was not randomized, we performed a propensity score–matched analysis to minimize potential confounding and selection bias between dTRA and conventional radial groups.

### PCI procedures, medications, and consent

All patients received dual antiplatelet therapy (DAPT) in accordance with the latest Japanese PCI guidelines. DAPT consisted of aspirin and a P2Y₁₂ inhibitor, administered with loading doses followed by 12 months of maintenance therapy. In patients requiring concomitant anticoagulation, triple therapy (anticoagulant plus DAPT) was restricted to the first month, after which anticoagulation combined with a single P2Y₁₂ inhibitor was continued per guideline recommendations. Dose reductions were permitted for patients at high bleeding risk at the operator’s discretion. GP IIb/IIIa inhibitors are not approved for clinical use in Japan; thus, none were administered in this study.

Procedural anticoagulation was initiated with a bolus of unfractionated heparin and adjusted based on activated clotting time (ACT), targeting ACT values > 250 s. The use of intracoronary imaging modalities and specific DAPT regimens were left to the operator’s discretion. According to the study protocol, all patients were intended to receive a biodegradable polymer-coated everolimus-eluting stent (Synergy™, Boston Scientific, MA, USA). Written informed consent was obtained from all patients prior to coronary angiography, angioplasty, and study enrollment. In cases where clinical instability precluded written consent, verbal consent was obtained from the patient or a legal representative and subsequently confirmed in writing after recovery.

### Endpoints

The primary endpoint of the current study was sheath-to-balloon time, in alignment with the objective of the parent SPEEDY PCI study, which focused on evaluating procedural efficiency from arterial access to revascularization. Given the well-established clinical importance of timely reperfusion in STEMI, door-to-balloon time was also assessed as a key secondary endpoint. Other secondary endpoints included PCI success, total ischemic time, contrast dye volume, radiation exposure dose, fluoroscopic time, length of hospital stay, total hospitalization fees, all-cause mortality, cardiac mortality, and bleeding events at 30-day and 1-year follow-up.

### Statistical analysis & ethics

Continuous variables were reported as means ± standard deviation and compared using Student’s t-test, assuming normal distribution. Categorical variables were expressed as counts and percentages and compared using the chi-square or Fisher’s exact test, as appropriate. A two-sided p-value < 0.05 was considered statistically significant.

In the analysis of the original cohort, significant differences were observed in baseline patient risk profiles such as Killip class and GRACE score. In addition, door-to-sheath (D2S) time—which strongly reflects institutional procedural responsiveness—also differed between groups. To correct for these three sources of bias, propensity score matching was performed. Matching was performed with the use of a 1:1 matching protocol without replacement (greedy-matching algorithm), with a caliper width equal to 0.2 of the standard deviation of the logit of the propensity score. As shown in the tables, propensity score matching generated a dataset cohort in which these three variables no longer showed significant differences. All statistical analyses were performed using SAS version 9.4 (SAS Institute, Cary, North Carolina).

The SPEEDY PCI trial was approved by the Tokai University Certified Review Board and conducted in accordance with the principles of the Declaration of Helsinki. The trial was registered at ClinicalTrials.gov (NCT05604976). The sponsor was not involved in the conduct, data collection, analysis, or manuscript preparation. This subanalysis was prespecified in the original trial protocol, and no additional ethical approval was required.

## Results

### Patient recruitment and assignment

A total of 430 patients were enrolled in the SPEEDY PCI study. After excluding 50 patients according to the study protocol, 380 patients were included in the final analysis of the parent study. For the present subanalysis, 10 patients treated via femoral or brachial access were excluded. Consequently, 370 patients who underwent PCI via either the distal radial (*n* = 63) or conventional radial (*n* = 307) approach were analyzed.

### Patient characteristics

**Fig. 1:**
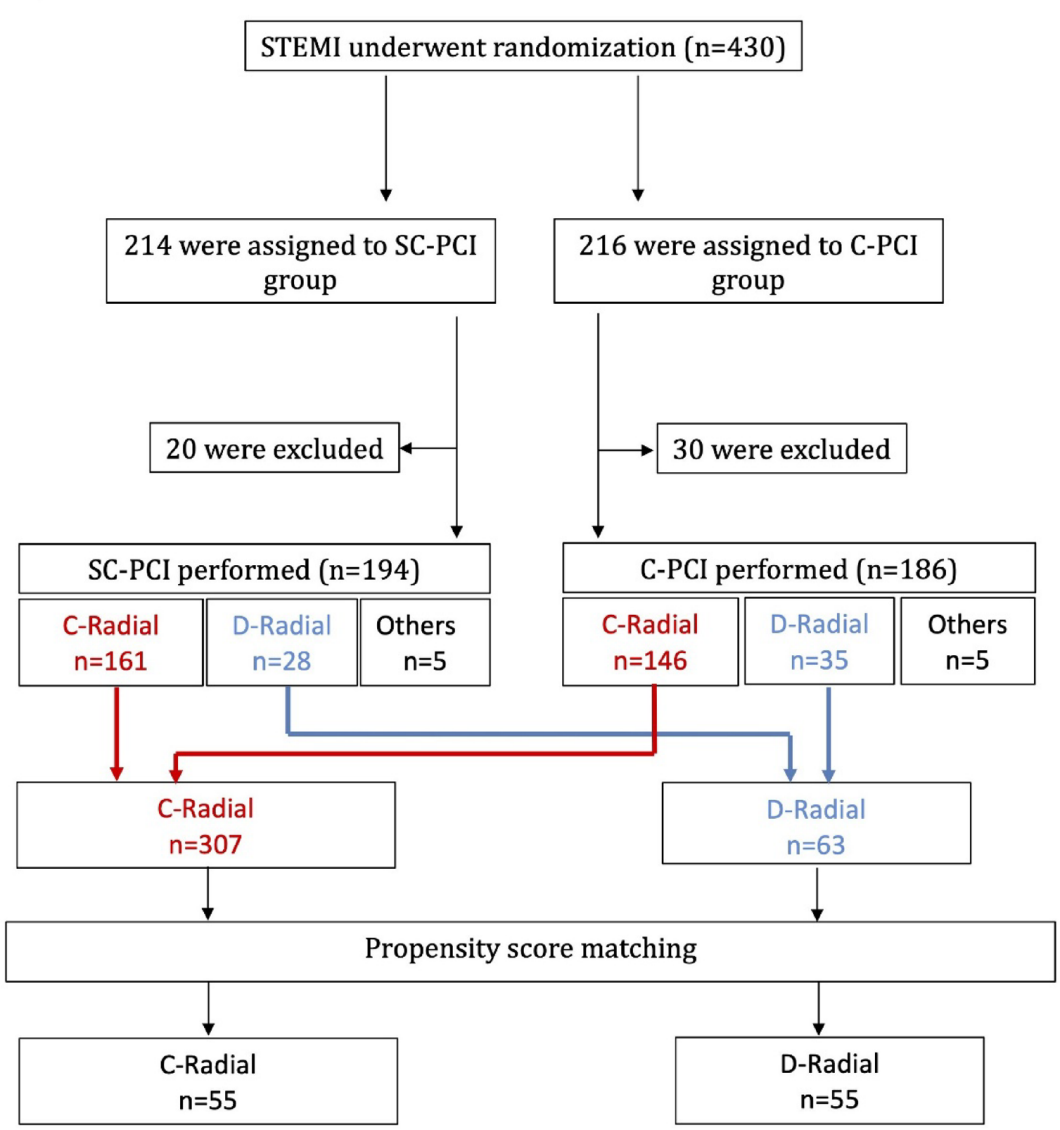
Flow diagram of patient inclusion and propensity score matching in the SPEEDY PCI subanalysis. In the parent SPEEDY study, 430 STEMI patients were randomized to single-catheter PCI (SC-PCI; *n* = 214) or conventional PCI (C-PCI; *n* = 216). After excluding 20 and 30 patients, respectively, the remaining 194 SC-PCI and 186 C-PCI patients with complete procedural data were included. For the present subanalysis, we focused on access routes. In the SC-PCI cohort (*n* = 194), access routes consisted of conventional radial (*n* = 161), distal radial (*n* = 28), and other access (*n* = 5). In the C-PCI cohort (*n* = 186), the corresponding access routes were conventional radial (*n* = 146), distal radial (*n* = 35), and other access (*n* = 5). Across both cohorts, conventional radial access totaled 307 cases, and distal radial access totaled 63 cases.Propensity-score matching identified 55 patients who underwent dTRA and 55 matched patients who underwent conventional radial access. C-Radial, conventional radial; D-Radial, distal radial.

In the original cohort (*n* = 370), patients undergoing the distal radial approach (dTRA) tended to present with lower blood pressure and higher Killip class, suggesting greater hemodynamic instability compared with those treated via the conventional radial approach. To reduce baseline imbalance between the two access groups, propensity score –matched analysis matching was performed using Killip class, GRACE score, and door-to-sheath time as covariates. After propensity score matching, 55 pairs were generated, achieving balance in the prespecified matching variables; however, several other clinical characteristics remained imbalanced, indicating residual differences between groups (Table 1; Fig. 1).

**Table 1:** Patient characteristics.

	Original cohort			Propensity matched cohort		
	Distal Radial Access (*n* = 63)	Radial Access (*n* = 307)	*p* value	Distal Radial Access (*n* = 55)	Radial Access (*n* = 55)	*p* value
Age - years	69 ± 14	69 ± 12	0.99	67 ± 13	73 ± 11	0.01
Male sex - no.(%)	52(82.5%)	253(82.4%)	0.98	46(84%)	41(75%)	0.24
Height - cm	165 ± 8	164 ± 8	0.68	165 ± 8	162 ± 8	0.05
Body weight - kg	65 ± 13	66 ± 13	0.63	65 ± 13	60 ± 12	0.04
BMI - kg/m²	24 ± 4	24 ± 4	0.48	24 ± 4	23 ± 3	0.17
Smoking never/current/past/unknown smoke - %	33/30/35/2	33/39/26/2	0.40	32/31/35/2	38/33/27/2	0.87
Diabetic mellitus - no.(%)	20(32%)	90(29%)	0.76	16(29%)	20(36%)	0.41
Dyslipidemia - no.(%)	48(76%)	196(64%)	0.15	45(82%)	37(67%)	0.17
Hypertension - no.(%)	39(62%)	219(71%)	0.24	34(62%)	40(73%)	0.22
Prior PCI - no.(%)	7(11%)	34(11%)	0.99	6(11%)	6(11%)	1
Prior CABG - no.(%)	0	0		0	0	
Old myocardial infarction - no.(%)	5(8%)	24(8%)	0.97	4(7%)	4(7%)	1
Prior ischemic stroke none/within 6 m/before 6 m/unknown, %	94/0/6/0	96/0.3/3/0.7	0.40	95/0/5/0	93/0/5/2	0.60
Prior hemorrhagic stroke - no.(%)	0	4(1.3%)	0.36	0	4(7%)	0.04
Prior ischemic stroke none/within 6 m/before 6 m/ before 12 m/unknown, %	100/0/0/0/0	96/ 0/0.3/ 0.7/ 3	0.51	100/0/0/0	98/0/0/2	0.32
Prior heart failure - no.(%)	1(1.6%)	0	0.03	1(1.8%)	0	0.32
Lower Extremity Artery Disease - no.(%)	3(4.8%)	6(2.0%)	0.14	2(3.6%)	1(1.8%)	0.31
History of cancer - no.(%)	2(3.2%)	8(2.6%)	0.80	2(3.6%)	3(5.5%)	0.65
Oral anticoagulant - no.(%)	2(3.2%)	12(3.9%)	0.78	1(1.8%)	4(7.3%)	0.17
High bleeding risk - no. (%)	25(40%)	93(30%)	0.31	18(33%)	29(53%)	0.10
Killip classification I/II/III/IV, %	52/ 32/ 11/ 5	86/ 12/ 2/ 1	< 0.0001	60/31/7/2	55/34/7/4	0.89
Systolic blood pressure, mmHg	127 ± 28	146 ± 27	< 0.0001	129 ± 27	140 ± 25	0.03
Diastolic blood pressure, mmHg	78 ± 18	89 ± 19	< 0.0001	80 ± 17	85 ± 17	0.09
Heart rate, /min	76 ± 17	79 ± 16	0.14	76 ± 17	82 ± 19	0.07
Creatinine, mg/dL	0.9 ± 0.4	0.9 ± 0.3	0.62	0.9 ± 0.3	1.0 ± 0.5	0.31
eGFR, ml/min./1.73 m²	67 ± 23	67 ± 20	0.88	70 ± 22	62 ± 18	0.06
Hemoblogin, g/dL	13.5 ± 2.2	14.3 ± 1.9	0.01	13.8 ± 2.2	14.8 ± 2.0	0.95
Platelet, /mL3	22.7 ± 6.2	22.8 ± 6.5	0.90	23.0 ± 5.9	21.8 ± 7.2	0.35
Grace score	171 ± 38	152 ± 29	0.0004	162 ± 32	171 ± 32	0.15
Off-hours presentation no. (%)	35 (55.6%)	149 (48.5%)	0.31	32 (58.2%)	27 (49.1%)	0.34

BMI, body mass index, STEMI, ST-elevation myocardial infarction; PCI, percutaneous coronary intervention; CABG, coronary artery bypass graft; eGFR, estimated glomerular filtration rate.

### Procedural characteristics

Procedural details are summarized in Table 2. In the matched cohort, access-site differences reflected the intrinsic nature of the two approaches—dTRA procedures were predominantly right-sided and frequently utilized smaller or sheathless systems, whereas the conventional radial approach mainly used 6 F sheaths. The number of diseased vessels, culprit lesion distribution, and stent implantation rates were comparable between groups. Although the dTRA group more often employed dedicated small-caliber catheters including sheathless guiding catheters, the overall procedural success rate of both groups was high and similar. No significant differences were observed in the use of mechanical circulatory support devices (Intra-aortic balloon pumping, Extra corporeal membrane oxygenation, Impella) or in periprocedural complications (Table 2).

**Table 2:** PCI procedure and device.

	Original cohort			Propensity matched cohort		
	Distal Radial Access (*n* = 63)	Radial Access (*n* = 307)	*p* value	Distal Radial Access (*n* = 55)	Radial Access (*n* = 55)	*p* value
Access site						
Left radial - no.(%)	0	46(15%)	< 0.0001	0	10(18%)	< 0.0001
Right radial - no.(%)	0	261(85%)		0	45(82%)	
Left distal radial - no.(%)	2(3%)	0		1(2%)	0	
Right distal radial - no.(%)	61(97%)	0		54(92%)	0	
Access change - no.(%)	3(4.8%)	7(2.3%)	0.27	2(3.6%)	2(3.6%)	1
Culprit lesion						
RCA - no.(%)	24(38.1%)	104(33.9%)	0.33	20(36%)	20(36%)	0.64
LAD - no.(%)	36(57.1%)	178(58.0%)		32(58%)	31(56%)	
LCX - no.(%)	2(3.2%)	24(7.8%)		2(4%)	4(7%)	
LM - no.(%)	1(1.6%)	1(0.3%)		1(2%)	0	
No. of vessel disease						
1 - no.(%)	35(55.5%)	205(66.8%)	0.22	33(60%)	36(65%)	0.79
2 - no.(%)	18(28.6%)	69(22.5%)		14(25%)	13(24%)	
3 - no.(%)	10(15.9%)	33(10.8%)		8(15%)	6(11%)	
Non-protected left main disease	1(1.6%)	8(2.6%)	0.63	1(2%)	2(4%)	0.56
No. of angiographic catheter	0.6 ± 0.6	0.9 ± 1.0	0.007	0.7 ± 0.6	1.0 ± 0.9	0.04
No. of guiding catheter	1.1 ± 0.3	1.1 ± 0.4	0.32	1.1 ± 0.4	1.1 ± 0.3	0.55
No. of total catheter	1.8 ± 0.8	1.9 ± 1.0	0.09	1.8 ± 0.7	2.0 ± 1.0	0.11
Guiding catheter shape			0.001			0.04
Ikari - no.(%)	44(69.8%)	210(68.4%)		39(71%)	40(73%)	
Judkins - no.(%)	1(1.6%)	47(15.3%)		1(2%)	8(14%)	
Amplatz - no.(%)	4(6.4%)	7(2.3%)		3(5%)	2(4%)	
VODA/EBU/XB - no.(%)	14(22.2%)	33(10.7%)		12(22%)	5(9%)	
Others - no.(%)	0	10(3.3%)		0		
Sheath size			< 0.0001			< 0.0001
sheathless - no.(%)	42(66.6%)	0		35(64%)	0	
5 F - no.(%)	1(1.6%)	3(1.0%)		1(1.8%)	1(1.8%)	
6 F - no.(%)	20(31.8%)	278(90.6%)		19(35%)	47(85%)	
7 F - no.(%)	0	26(8.4%)		0	7(13%)	
Guiding catheter size						
5 F - no.(%)	1(1.6%)	2(0.7%)	0.08	1(2%)	0	0.08
6 F - no.(%)	62(98.4%)	284(92.5%)		54(98%)	51(93%)	
7 F - no.(%)	0	21(6.8%)		0	4(7%)	
Guiding catheter shaft						
Mach1 - no.(%)	0	29(9.4%)	< 0.0001	0	5(9%)	< 0.0001
Heartrail2 - no.(%)	16(25.4%)	183(59.6%)		16(29%)	35(64%)	
Launcher - no.(%)	0	13(4.2%)		0	1(2%)	
Taiga - no.(%)	5(7.9%)	2(0.7%)		3(5%)	0	
Profit - no.(%)	1(1.6%)	10(3.3%)		1(2%)	2(4%)	
Hyperion - no.(%)	41(65.1%)	69(22.5%)		35(64%)	12(22%)	
Others - no.(%)	0	1(0.3%)		0	0	
Coronary stent implantation	58(92.1%)	276(89.9%)	0.60	50(91%)	47(85%)	
Synergy stent - no.(%)	51(81.0%)	245(79.8%)	0.001	43(78%)	40(73%)	0.08
Other drug eluting stents - no.(%)	7(11.1%)	31(10.1%)		7(13%)	7(13%)	
Intentional stentless - no.(%)	2(3.2%)	29(9.4%)		2(4%)	8(15%)	
Stent delivery failure - no.(%)	0	2(0.7%)		0	0	
Others - no.(%)	3(4.8%)	0		3(5%)	0	
Post-PCI pacemaker - no.(%)	1(1.6%)	6(2.0%)	0.85	1(1.8%)	1(1.8%)	1
Post-PCI IABP - no.(%)	5(7.9%)	13(4.2%)	0.21	3(5%)	4(7%)	0.69
Post-PCI ECMO - no.(%)	1(1.6%)	3(1.0%)	0.66	0	1(1.8%)	0.32
Post-PCI Impella - no.(%)	0	1(0.3%)	0.65	0	0	0
Randomization to SC-PCI group	28(44%)	161(52%)	0.25	24(44%)	25(45%)	0.85

RCA, right coronary artery; LAD, left anterior descending artery; LCX, left circumflex artery; LM, left main artery, IABP, intra aortic balloon pumping; ECMO, extra corporeal membrane oxygenation.

### PCI results

As shown in Table 3, key procedural time metrics were significantly shorter in the dTRA group. Specifically, in the matched cohort, sheath-to-balloon time (12.5 ± 11.5 vs. 19.7 ± 11.7 min, *p* = 0.002) and door-to-balloon time (50.2 ± 25.9 vs. 62.3 ± 19.9 min, *p* = 0.007) remained shorter in the dTRA group, suggesting procedural efficiency independent of baseline confounders. Door-to-sheath time was similar between groups (37.7 ± 22.5 vs. 42.6 ± 18.6 min, *p* = 0.22), indicating that workflow differences rather than access technique likely contributed to disparities in the unmatched cohort. Fluoroscopic time was significantly shorter (15.6 ± 11.3 vs. 26.9 ± 13.6 min, *p* < 0.0001), and radiation exposure was lower in the dTRA group (1068 ± 690 vs. 1433 ± 927 mGy, *p* = 0.02). PCI success and final TIMI 3 flow rates were similarly high in both groups (> 90%). The length of ICU stay was longer in the dTRA group (3.8 ± 1.8 vs. 3.6 ± 5.0 days; *p* = 0.78), while length of hospital stays, total hospitalization fees, and peak CK values showed no significant differences between the two groups (Table 3). Supplemental data from the SPEEDY PCI study demonstrated that door-to-balloon time was significantly longer among patients presenting during off-hours in the overall cohort (67.2 ± 37.5 min vs. 60.2 ± 24.2 min, *p* = 0.03; off-hours *n* = 186, on-hours *n* = 184) (Table 4). The proportion of off-hours presentations was similar between the dTRA and conventional radial groups (Table 1).

**Table 3:** PCI results.

	Original cohort			Propensity matched cohort		
	Distal Radial Access (*n* = 63)	Radial Access (*n* = 307)	*p* value	Distal Radial Access (*n* = 55)	Radial Access (*n* = 55)	*p* value
PCI success - no.(%)						
Successful dilatation	62(98.4%)	301(98.1%)	0.85	54(98.2%)	53(96.4%)	0.56
Successful dilatation and final TIMI3	59(93.7%)	281(91.5%)	0.57	51(92.7%)	47(85.5%)	0.22
Sheath to balloon time, min	12.5 ± 11.9	17.9 ± 9.8	0.001	12.5 ± 11.5	19.7 ± 11.7	0.002
Door to sheath time, min	35.6 ± 21.8	48.9 ± 31.0	< 0.0001	37.7 ± 22.5	42.6 ± 18.6	0.22
Door to balloon time, min	48.1 ± 25.5	66.8 ± 31.9	< 0.0001	50.2 ± 25.9	62.3 ± 19.9	0.007
Onset to door time, min	164.2 ± 147.9	175.0 ± 147.8	0.60	160.7 ± 152.6	170.5 ± 129.9	0.72
Onset to balloon time, min	212.2 ± 152.9	241.8 ± 151.2	0.16	210.9 ± 157.6	232.8 ± 127.7	0.43
Total ischemic time, min	215.8 ± 157.6	248.1 ± 153.8	0.15	213.7 ± 162.8	230.9 ± 122.5	0.55
Fluoroscopic time, min	17.7 ± 17.6	24.4 ± 16.4	0.006	15.6 ± 11.3	26.9 ± 13.6	< 0.0001
Fluoroscopic dose, mGy	1157 ± 967	1155 ± 868	0.99	1068 ± 690	1433 ± 927	0.02
Contrast dye volume, mL	121 ± 42	134 ± 54	0.03	122 ± 44	132 ± 52	0.29
ICU stay, days	4.4 ± 3.2	3.0 ± 3.1	0.002	3.8 ± 1.8	3.6 ± 5.0	0.78
Hospital stay, days	13.2 ± 6.2	11.9 ± 8.5	0.17	12.1 ± 4.9	13.6 ± 11.9	0.38
Cost, million Yen	1.75 ± 0.75	1.83 ± 1.06	0.49	1.66 ± 0.59	2.10 ± 1.76	0.08
Peak CK, IU/L	2461 ± 2149	2715 ± 2511	0.41	2409 ± 1761	2721 ± 2197	0.41

PCI, percutaneous coronary intervention; ICU, intensive care unit.

**Table 4:** Patient characteristics (presentation hours).

	Original cohort (*n* = 370)			After propensity matching (*n* = 110)		
	On-hours presentation (*n* = 186)	Off-hours presentation (*n* = 184)	*p* value	On-hours presentation (*n* = 51)	Off-hours presentation (*n* = 59)	*p* value
Door to balloon time (min)	60.2 ± 24.2	67.2 ± 37.5	0.03	56.1 ± 22.4	56.4 ± 25.4	0.95

### Clinical outcomes

Clinical outcomes at 30 days and 1 year are summarized in Table 4. At 30 days, again in the matched cohorts, all-cause mortality (1.8% vs. 7.2%, *p* = 0.30), cardiac death, stroke, and major bleeding (BARC ≥ 3) did not differ significantly between groups. No recurrent myocardial infarctions were observed. At 1 year, mortality remained comparable (5.5% vs. 9.1%, *p* = 0.44), and there were no significant differences in cardiac or non-cardiac deaths, stroke, or major bleeding. No recurrent myocardial infarctions were observed (Table 5).

**Table 5:** Clinical outcome at 30 days and 1year.

	Original cohort			Propensity matched cohort		
	Distal Radial Access (*n* = 63)	Radial Access (*n* = 307)	*p* value	Distal Radial Access (*n* = 55)	Radial Access (*n* = 55)	*p* value
30 days						
All-cause mortality - no.(%)	3(4.8%)	6(2.0%)	0.35	1(1.8%)	4(7.2%)	0.30
Cardiac death - no.(%)	2(3.2%)	3(1.0%)		1(1.8%)	2(3.6%)	
Non-cardiac death - no.(%)	1(1.6%)	3(1.0%)		0	2(3.6%)	
Recurrent MI - no.(%)	0	0		0	0	
Stroke - no.(%)	2(3.2%)	3(1.0%)	0.19	2(3.6%)	0	0.15
Hemorrhagic - no.(%)	0	1(0.3%)		0	0	
Ischemic - no.(%)	2(3.2%)	2(0.7%)		2(3.6%)	0	
Bleeding (BARC 3 or 5) - no.(%)	0	4(1.3%)	0.66	0	2(3.6%)	0.36
PCI Puncture site - no.(%)	0	0		0	1(1.8%)	
ECMO Puncture site - no.(%)	0	1(0.3%)		0	0	
GI tract - no.(%)	0	1(0.3%)		0	0	
Intracranial hemorrhage - no.(%)	0	1(0.3%)		0	1(1.8%)	
Cardiac perforation - no.(%)	0	1(0.3%)		0	0	
1 year						
All-cause mortality - no.(%)	5(7.9%)	9(3.0%)	0.06	3(5.5%)	5(9.1%)	0.44
Cardiac death - no.(%)	2(3.2%)	5(1.7%)	0.41	1(1.8%)	3(5.6%)	0.30
Non-cardiac death - no.(%)	3(4.9%)	4(1.3%)	0.07	2(3.6%)	2(3.7%)	0.95
Recurrent MI - no.(%)	0	2(0.7%)	0.43	0	0	
Stroke - no.(%)	3(5.0%)	5(1.7%)	0.12	3(5.5%)	1(1.8%)	0.33
Hemorrhagic - no.(%)	1(1.8%)	3(1.0%)	0.65	1(1.8%)	1(1.8%)	
Ischemic - no.(%)	2(3.2%)	2(0.7%)	0.08	2(3.6%)	0	
Bleeding (BARC 3 or 5) - no.(%)	0	5(1.7%)	0.31	0	2(3.6%)	0.15

MI, myocardial infarction; BARC, bleeding academic research consortium; PCI, percutaneous coronary intervention; ECMO, extra corporeal membrane oxygenation; GI tract, gastrointestinal tract.

## Discussion

This prespecified subanalysis of the SPEEDY PCI study demonstrates that the distal radial approach (dTRA) is both feasible and efficient for primary PCI in patients presenting with STEMI. Among unmatched cohorts, patients in the dTRA group presented with worse baseline clinical status—including higher Killip class, lower blood pressure, and elevated GRACE scores—than those in the conventional radial group. After adjustment, procedural outcomes such as sheath-to-balloon and door-to-balloon times remained significantly shorter in the dTRA group, while clinical outcomes, including 30-day mortality and major bleeding events, were comparable.

In 2017, Kiemeneij published the first report highlighting the potential benefits of dTRA^4^. The study demonstrated its feasibility for angiography and PCI, reporting no cases of forearm radial artery occlusion (RAO), no major bleeding events, and a reduction in hemostasis time. Importantly, the study included six patients with STEMI and 17 with NSTEMI, suggesting the potential applicability of this approach in patients with acute coronary syndrome (ACS). Since then, one of the key objectives for interventional cardiologists has been to validate the feasibility and efficacy of the distal radial approach in ACS—particularly in STEMI patients—as several studies have suggested that this approach may be less invasive than the conventional radial approach, offering lower access site complication rates and improved patient comfort^5^^6^^7^. In addition, a recent randomized controlled trial in patients undergoing elective PCI reported a remarkably low RAO rate of 0.3% in the dTRA group, with no BARC type 3–5 bleeding complications and shorter hemostasis times compared to the conventional radial approach^12^.

Despite these potential advantages, performing primary PCI via the distal radial approach can be technically demanding especially in emergency settings due to the smaller vessel diameter. Indeed, initial experience from Kiemeneij noted a puncture failure rate of 11.4% (8 out of 70 patients)^4^. Further study examining the learning curve for achieving technical proficiency demonstrated that approximately 200 procedures were required to reach a stable success rate of > 94%^13^. Thus, operator expertise has been identified as an important determinant of procedural success in PCI with dTRA and may even contribute to reducing major complications^14^. Likewise, ultrasound guidance serves as a useful adjunct to improve puncture success and procedural safety, even in patients with hypotension or cardiogenic shock^15^. Furthermore, in a single-center study involving 109 STEMI patients treated via distal radial, conventional radial, and femoral approaches, 42 patients were initially intended to be treated with dTRA, of whom 35 were successfully treated using dTRA. The remaining 7 patients failed since 5 experienced puncture failure, and 2 had artery spasm^16^. Another single center, score matched study in patients with acute chest pain showed that the cannulation success rate in the dTRA group was significantly lower than in the conventional radial approach group (87.4% vs. 94.8% respectively, *p* < 0.05). However, the dTRA group had a significantly shorter hemostasis duration [4 (4, 4) hours vs. 10 (8, 10) hours, *p* < 0.001] and a lower incidence of minor bleeding (BARC Type I and II) (0.85% vs. 5.48%, *p* = 0.045). A STEMI-specific subgroup analysis within the same study revealed no significant differences in puncture time, door-to-balloon time, or total procedure time between the two group^17^. More recently, a separate single-center study from Japan reported a door-to-balloon time for the dTRA of 40.0 ± 30.8 min (*n* = 77), which was compared with a historical control from the same hospital (*n* = 103), with a time of 42.6 ± 31.6 min (*p* = 0.52)^18^.

In the current study, both sheath-to-balloon and door-to-balloon times remained significantly shorter in the dTRA group even after propensity score–matched analysis. To better interpret these time differences, it is important to consider potential confounding factors that may affect procedural timing. Procedural time metrics, in particular door-to-balloon time are also known to be influenced by external factors—particularly the timing of patient presentation (on-hours vs. off-hours)^19,20^. Therefore, we conducted an exploratory analysis using supplemental data from the parent SPEEDY PCI study. Consistent with previous reports, door-to-balloon time was significantly longer in patients presenting during off-hours compared with those presenting during on-hours in the original cohort (Table 4). However, the proportion of off-hours presentations did not differ significantly between the dTRA and conventional radial groups, both before and after propensity matching (Table 1), suggesting that off-hours arrival was unlikely to have biased the comparison between access strategies. Furthermore, in the matched cohort, dTRA procedures were predominantly right sided and frequently utilized smaller or sheathless systems, whereas the conventional radial approach mainly employed 6 F sheaths. These intrinsic differences in procedural strategy likely reflect operator familiarity and institutional workflow optimization and may themselves contribute to shorter reperfusion times. As such, the observed efficiency in the dTRA group cannot be ascribed solely to the access site; instead, it likely represents the combined effect of access selection, device strategy including preference for sheathless catheter, and accumulated operator expertise.

Taken together, these findings indicate that the observed procedural efficiency of dTRA likely reflects not only the technical feasibility of the approach but also institutional workflow optimization and accumulated operator expertise. Accordingly, while dTRA appears safe and efficient when performed by experienced operators, its broader implementation should be guided by institutional readiness and appropriate operator training. Importantly, despite the procedural differences, clinical outcomes—including mortality and bleeding events—were comparable between groups, supporting the overall safety of dTRA in STEMI care.

### Limitations

Several limitations of this study should be acknowledged. First, although access site selection (distal vs. conventional radial) in the original SPEEDY PCI study was left to the operator’s discretion, rendering this subanalysis observational in nature, propensity score matching was performed to reduce baseline confounding. However, residual bias related to operator or institutional experience cannot be fully excluded and may have contributed to the shorter procedural times observed in the dTRA group. Furthermore, the shorter reperfusion times did not translate into improved 30-day outcomes, underscoring the need for caution when interpreting procedural advantages in isolation. Second, despite matching on three major covariates (Killip class, GRACE score, and door-to-sheath time), residual confounding from unmatched variables—especially age, one of the strongest predictors of clinical outcomes, as well as body weight and systolic blood pressure—remained and may have influenced the results. In addition, the substantially higher use of the sheathless technique in the dTRA group (64%) represents a potential confounder, as this method may inherently influence procedural time independent of access site^21^. Third, the present study did not assess coronary anatomical complexity (e.g., SYNTAX Score II), which might have influenced procedural and short-term clinical outcomes. Fourth, protocol-driven procedural variations—such as the use of a single universal guiding catheter versus sequential diagnostic and guiding catheters—introduced variability in revascularization time. Fifth, approximately half of all procedures in the SPEEDY PCI study were performed using the Ikari-left catheter; therefore, generalizability to centers using different guiding catheters may be limited. Sixth, the study did not account for operator expertise or learning curves related to dTRA and the single-catheter technique, which may have influenced procedural efficiency. Finally, although the SPEEDY PCI trial was conducted during the COVID-19 pandemic, detailed data on infection-control policies and their potential impact on procedural time metrics were not collected. Such measures—such as decisions regarding whether to proceed with primary PCI before antigen-test confirmation—may have influenced workflow and clinical outcomes.^22,23^.

## Conclusion

In this prespecified subanalysis of the multicenter SPEEDY PCI study, the distal radial approach was feasible and could be performed safely in patients with STEMI by experienced operators. However, due to residual confounding and both patient-related and procedural heterogeneity—including differences in age and body weight, the predominant use of sheathless systems, and other procedural variations in the dTRA group—the present analysis does not allow firm conclusions regarding comparative efficacy between access strategies. Further randomized studies using standardized procedural protocols are needed to clarify this issue.

## Data Availability

The datasets used and/or analyzed during the current study are available from the corresponding author on reasonable request.
